# The Association Between Age of First Exposure to American Football at a Young Age and Later-Life Health Issues in Healthy, Community-Dwelling Adults

**DOI:** 10.1007/s40279-025-02239-w

**Published:** 2025-05-14

**Authors:** Grant H. Rigney, John E. Dugan, Anthony Bishay, Soren Jonzzon, Jacob Jo, Kristen L. Williams, Scott L. Zuckerman, Douglas P. Terry

**Affiliations:** 1 Harvard Medical School, Boston, MA USA; 2 Vanderbilt Sport Concussion Center, Vanderbilt University Medical Center, Nashville, TN USA; 3 The University of Tennessee Health Science Center, Memphis, TN USA; 4 Vanderbilt University School of Medicine, Nashville, TN USA; 5 Department of Neurological Surgery, Vanderbilt Sport Concussion Center, Vanderbilt University Medical Center, 1500 21st Ave S, Village at Vanderbilt, Suite 4300, Nashville, TN 37212 USA; 6 Johns Hopkins University, Department of Neurosurgery, Baltimore, MD USA

## Abstract

**Introduction:**

Younger age of first exposure (AFE) to American Football (football) is associated with later-life health problems among former professional athletes in several studies; however, studies examining amateur (i.e., nonprofessional) athletes are less clear.

**Objective:**

In a cohort of former amateur American Football players, this study assessed whether AFE to football was associated with: (1) psychiatric and neurobehavioral symptoms, (2) cognitive difficulties, (3) general health problems, (4) motor symptoms, and (5) functional status.

**Methods:**

A cross-sectional survey study was conducted using the ResearchMatch platform. The key independent variable was age of first exposure to football (AFE < 12 versus AFE ≥ 12). Main outcomes included depressive symptoms (Patient Health Questionnaire-9; PHQ-9), anxiety symptoms (Generalized Anxiety Disorders-7; GAD-7), cognitive difficulties (British Columbia Cognitive Complaints Inventory; BC-CCI), Neurobehavioral Symptom Inventory (NSI) score, and prevalence of other health problems. Multivariable regressions were assessed for associations between AFE and outcome variables.

**Results:**

In total, 107 male participants with exposure to football (mean age: 60.6 ± 15.1 years) reported an average of 4.2 ± 2.7 years of exposure to football, with an average AFE of 11.7 ± 3.1 years. In multivariable analyses, AFE < 12 was not a significant predictor of PHQ-9 (unstandardized beta, *B*: 0.51, standard error, SE: 1.25, *p* = 0.682), GAD-7 (*B*: 0.09, SE: 0.95, *p* = 0.926), NSI (*B*: − 0.56, SE: 2.93, *p* = 0.850), or BC-CCI (*B*: − 0.65, SE: 0.77, *p* = 0.403). However, more prior concussions were associated with worse PHQ-9 (*B*: 0.44, SE: 0.10, *p* < 0.001), GAD-7 (*B*: 0.33, SE: 0.07, *p* < 0.001), NSI (*B*: 1.04, SE: 0.23, *p* < 0.001), and BC-CCI scores (*B*: 0.26, SE: 0.06, *p* < 0.001). AFE < 12 did not predict general health problems or independent functional status.

**Conclusions:**

AFE to football was not associated with adverse psychiatric, cognitive, neurobehavioral, or general health outcomes among young, former amateur American Football players. However, more lifetime concussions were associated with adverse cognitive and psychiatric health outcomes. Future studies should examine similar outcomes in older cohorts with more comorbidities to further minimize potential confounding between general health and lack of later-life symptoms.

**Supplementary Information:**

The online version contains supplementary material available at 10.1007/s40279-025-02239-w.

## Key Points

**Table:** 

For amateur athletes, starting to play American Football before the age of 12 was not related to levels of depression, anxiety, cognitive difficulties, behavioral issues, or general health outcomes among relatively healthy later-life community-dwelling adults.
Higher levels of depression, anxiety, cognitive difficulties, and behavioral issues were significantly associated with increasing number of total lifetime concussions.
Individuals who started playing football before the age of 12 years did not have significantly more concussions than those who started play at or after the age of 12 years.

## Introduction

Examining early exposure to head impacts via contact sports, such as American Football (football), and how they may relate to long-term health outcomes has become an important area of research that may have direct impacts on public attitudes towards youth participation in football and other contact sports [1–3]. For example, a recent study revealed that a significant percentage of parents have reservations about allowing their 10-year-old children to participate in contact sports, citing concerns about the safety of head impacts and potential long-term cognitive consequences [4]. In line with these concerns, youth sports leagues and governing organizations have taken steps to address the issue, such as discussion about implementing age-related rules and guidelines to reduce head impact exposure and thus mitigate potential risks for young athletes [5–9].

Prior research studying the age of first exposure (AFE) to football has yielded diverse and incongruous results. For example, a hallmark study by Stamm et al. showed that former National Football League (NFL) players with an earlier AFE (i.e., AFE < 12 years) had worse cognitive functioning compared with their counterparts with a later AFE [10]; however, this study included a small sample size of individuals (*n* = 42) who were all symptomatic at presentation, limiting its generalizability. Critiques of this paper were published [11] and focused on the study’s lack of proper control variables that could explain the measured differences that the authors attributed to head impacts, the authors’ definition of concussion (e.g., “seeing stars” was deemed a concussion), inaccurate conclusions reported from cited studies, the use of older indices, and their lack of separation of football-related head impacts from other sport-related head impacts. Despite this, subsequent imaging studies from the same research group have shown that former NFL players with younger AFE had white matter differences in the corpus callosum [12], less cortical thickness [13], and smaller thalamic volume [14] compared with those with older AFE. Furthermore, studies from other high contact sports have shown similar associations between AFE and later-life cognitive functioning. Bryant et al. studied current and retired professional fighters and found that earlier AFE is associated with decreased psychomotor and processing speeds as well as smaller hippocampal and corpus callosum volumes bilaterally [15, 16]. Similarly, Zhuang et al. studied longitudinal cognitive trajectories among professional fighters who transition to inactive fighting status versus those who remain as active fighters and found that those who stopped fighting had significantly improved cognitive functioning levels over time compared with those who continued fighting [17].

In contrast, other studies have revealed no such associations in athletes from a variety of age groups and level of play. Studies examining the effects of AFE in collegiate athletes playing contact and/or noncontact sports found that earlier AFE did not lead to worse depressive symptoms or heightened psychological distress [18], longer symptom recovery, worse cognitive functioning, increased psychological distress post-concussion [19], balance deficits [20], neurocognitive functioning, or subjectively reported symptoms [21]. Several studies have also examined the effects of AFE on later-in-life brain health in individuals who played high school football and found no association between earlier AFE and later-in-life anxiety, depression, memory loss, chronic pain, headaches, anger, concentration problems, or neck pain [22, 23]. Further, two studies examining the effects of AFE in former professional athletes (e.g., NFL players) found that earlier AFE to football was not associated with worse neuroradiological, neurological, or neuropsychological health [24]; however, the second study did report a weak association between earlier AFE and anxiety, depression, sleep disturbances, pain, and emotional-behavioral dyscontrol [25]. Although the vectors of impact may be different in different sports, research on the effects of AFE on later-life cognitive symptoms in similar contact sports such as boxing has suggested that AFE does play a significant role in later-life hippocampal volumes, processing speed, and executive function [15]. The studies described above demonstrate incongruencies in the potential effects of AFE on young and healthy populations compared with older, symptomatic individuals, thus highlighting the need for additional research to clarify the effects of AFE in amateur football.

Research on the impact that AFE to football has on long-term brain health outcomes of amateur athletes remains sparse. To address this gap, the current study examined a cohort of community-dwelling men who had a history of participation in football. The primary objective was to investigate the potential association between an earlier AFE to football and adverse brain health outcomes as participants age, specifically focusing on the following domains: (1) psychiatric and neurobehavioral symptoms, (2) cognitive difficulties, (3) general health problems, (4) motor symptoms, and (5) functional status.

## Methods

### Participants

A cross-sectional study was conducted through ResearchMatch, a national volunteer registry that includes data on concussion history. ResearchMatch is funded by the US National Institutes of Health (NIH) Clinical Translational Science Award program and was created to be a comprehensive survey platform by multiple academic institutions. The only inclusion criterion for being on the registry is having an age of at least 18 years. The study title was “Assessing Brain Health in Adults” in ResearchMatch. Materials about this study provided to participants did not mention brain injury, sports, concussion, or similar terms in an effort to reduce participation bias. Participants were not financially compensated for participation in this study.

### Procedures

Study participants were contacted via email and given the opportunity to complete a survey online. Anyone over the age of 18 years was eligible to complete the survey. The current study only included men who endorsed playing American football at any level for at least 1 year. Participants who did not complete all sections of the survey and those with biologically implausible responses (e.g., Rett syndrome, myelomeningocele, or amyotrophic lateral sclerosis) were excluded. Further, some participants endorsed having conditions that are known to have significant cognitive, psychological, and behavioral symptoms (e.g., experiencing a concussion in the past 6 months, multiple sclerosis, Parkinson’s disease, dementia, and epilepsy). Participants who reported any one or more of these conditions were excluded to reduce the likelihood of erroneously attributing the known symptoms of these conditions to their football history.

### Exposure Variables

The primary exposure variable was age of first exposure (AFE) to organized football, reported by each participant. AFE was then dichotomized (< 12 years versus ≥ 12 years) and used as a binary variable, in line with multiple prior studies [1, 10, 12, 18, 21–23, 23, 26–28]. In addition, each participant recorded the age at which they stopped playing and the total years of football played during their lifetime. Similarly, they repeated this process for each other contact sport (i.e., soccer, hockey, rugby, lacrosse, martial arts, boxing, or wrestling). All participants also completed questions on basic demographic variables and the number of concussions they have experienced throughout their lifetime. The following definition of concussion was provided in the survey: “We define a concussion as a blow to the head or whiplash that caused any one or more of the following: (1) witnessed loss of consciousness (being ‘knocked out’ and someone seeing it), (2) loss of memory for events immediately before and/or after the injury, or (3) feeling dazed and confused for at least 30 s.” Participants were asked to report on the number of concussions they experienced throughout their life, as well as the date of their most recent concussion.

### Outcome Measures

The primary outcome variables were assessed via self-reported surveys and housed within the ResearchMatch platform and included (1) psychiatric and neurobehavioral symptoms, (2) cognitive difficulties, (3) general health problems, (4) motor symptoms, and (5) functional status. To measure current symptoms of depression, the Patient Health Questionnaire-9 (PHQ9) [29] was used, which includes nine questions about depressive symptoms over the preceding 2 weeks (range 0–27). Symptoms of anxiety were assessed via the General Anxiety Disorder-7 (GAD7) questionnaire [30], a seven-item self-reported survey (range 0–21), with higher scores indicating more symptoms. A cutoff score of ≥ 10 on the PHQ-9 and GAD-7 was operationalized as screening positively for current depression and current anxiety, respectively, consistent with prior studies [31, 32]. To measure prior histories of psychiatric disorders, patients were asked if they had ever been told by a healthcare professional that they have (or had) depression, anxiety, posttraumatic stress disorder, and/or substance use. Answers were recorded in a binary yes/no format. The Neurobehavioral Symptom Inventory (NSI) was used to assess the severity of somatic, sleep, cognitive, and psychological symptoms [33–37]. The questionnaire measures each symptom over the past 2 weeks on a five-point Likert scale, with higher scores indicating more symptoms (22 questions, score range 0–88). The British Columbia Cognitive Complaints Inventory (BC-CCI) [38–40] was used to assess perceived cognitive difficulties. This six-item scale asks participants to rate the severity of various cognitive problems experienced over the preceding week. The BC-CCI total scores range from 0 to 18, with higher scores indicating worse cognitive difficulties.

General health problems were assessed via asking participants about sleep problems (trouble falling or staying asleep), chronic pain, and migraines in the past year. Participants’ motor problems were assessed by asking about symptoms of dysarthria, ataxia, imbalance, or tremor over the preceding year. For each problem, participants could choose from the following options: never, rarely, sometimes, often, always. Problems were coded as present (i.e., yes: 1, no: 0) if the participant reported having problems “sometimes,” “often,” or “always.” In addition, functional status was self-assessed using the follow response options: independent, slightly reduced performance/functioning, definite impairment, not independent, and cannot participate (see Supplementary Table 1 for specific functional status question). Of note, difficulties in functional status could be related to any reason, including physical, cognitive, or psychological etiologies.

### Statistical Analysis

Descriptive statistics were performed on the entire sample as well as stratified by AFE < 12 versus AFE ≥ 12. Independent samples *t* tests were used to compare the two AFE groups on the basis of current age, number of prior concussions, and the total years of football exposure. Chi-squared tests were used to assess differences in the percentage of patients who reported having general health problems, motor problems, functional status, cognitive, and neurobehavioral issues in each group. A series of four separate multivariable linear regressions were performed to assess the association between AFE (binary) and total scores on the PHQ-9, GAD-7, NSI, and BC-CCI, adjusting for age, number of total concussions, and total years of football exposure. A series of five separate logistic regressions were performed to assess the association between AFE and sleep problems, chronic pain problems, migraines, motor problems, and functional status, when adjusting for age, number of total concussions, and total years of football exposure. Effect sizes were calculated in Cohen’s *d* (for *t* tests) and V/Phi (for Chi-squared). Effect sizes were interpreted on the basis of prior literature, as small (*d* = 0.2), medium (*d* = 0.5), and large (*d* = 0.8) for Cohen’s *d* [41] and as weak (V/Phi > 0.05), moderate (V/Phi > 0.10), and strong (V/Phi > 0.15) for V/Phi [42]. Analyses were completed using SPSS 28.0 [43].

### Ethical Considerations

The study was approved by the Vanderbilt Institutional Review Board (IRB, no. 230651).

## Results

### Sample Characteristics

Of the 1766 unique participants who consented to the study, individuals who completed less than 100% of the survey, including important variables of interest such as sport history, were excluded from all analyses (*n* = 232). Next, individuals who did not report their sex at birth as male were excluded (*n* = 861), as well as those who reported no exposure to football (*n* = 554). Additionally, quality control analyses revealed several participants (*n* = 4) who appeared to respond in an invalid manner and who were excluded (i.e., endorsed having multiple implausible conditions, such as myelomeningocele, Rett’s disorder, or amyotrophic lateral sclerosis). Last, those who reported having multiple neurological conditions (e.g., multiple sclerosis, Parkinson’s disease, epilepsy, dementia, or chronic traumatic encephalopathy), the symptoms of which would confound the outcomes being measured, were excluded (*n* = 8; 3/8 reported Parkinson’s disease, 2/8 reported epilepsy, 1/8 reported having multiple sclerosis, and 2/8 reported having dementia), leaving 107 participants that were analyzed.

The 107 participants had a mean age of 60.6 years (median 64, standard deviation, SD 15.1, 100% men). The vast majority of the sample was white (87.9%; Black/African American: 10.3%; other: 1.9%), and 96.3% was not Hispanic or Latino (Hispanic or Latino/a: 3.7%). Most of the sample had a college education or greater (70.1%). About one-fourth (27.1%, *n* = 29) reported having no history of concussion, 15.9% (*n* = 17) reported having one prior concussion, 18.7% (*n* = 20) reported having two prior concussions, and 37.4% (*n* = 40) reported having three or more prior concussions. Participants reported an average of 4.2 (SD 2.7) years of exposure to football. The average AFE to football was 11.65 years (median 12.0, SD 3.1). The majority of the sample had an AFE to football ≥ 12 years (61.7% versus 38.3% in AFE < 12 years group), 81.3% (*n* = 87/107) played football at least through high school, 12.1% (*n* = 13/107) played at least through college, and the remainder (6.6%, *n* = 7) stopped playing football before high school (Table 1). In terms of years of exposure to football, 52 individuals played < 4 years (i.e., 1–3 years), 52 played between 4–10 years, and 3 individuals played 11 or more years.

**Table 1:** Demographic characteristics of study participants separated by AFE to American Football

Demographics	Total sample, *N* = 107	AFE < 12 (*N* = 41)	AFE ≥ 12 (*N* = 66)
Age, years	*M* = 60.6, Md = 64, IQR 49.0–73.0, SD 15.1, range 64	*M* = 53.0, Md = 52, IQR 44.5–62.5, SD 13.4, range 52	*M* = 65.3, Md = 69, IQR 60.5–75.0, SD 14.2, range 64
Race			
Black/African American	*n* = 11 (10.3%)	*n* = 8 (19.5%)	*n* = 3 (4.5%)
White	*n* = 94 (87.9%)	*n* = 32 (78.0%)	*n* = 62 (93.9%)
Other	*n* = 2 (1.9%)	*n* = 1 (2.5%)	*n* = 1 (1.5%)
Ethnicity			
Hispanic or Latino	*n* = 4 (3.7%)	*n* = 1 (2.4%)	*n* = 3 (4.5%)
Not Hispanic or Latino	*n* = 103 (96.3%)	*n* = 40 (97.6%)	*n* = 63 (95.5%)
Marital status			
Married	*n* = 67 (62.6%)	*n* = 21 (51.2%)	*n* = 46 (69.7%)
Living with partner	*n* = 3 (2.8%)	*n* = 2 (4.9%)	*n* = 1 (1.5%)
Separated/divorced	*n* = 16 (15.0%)	*n* = 7 (17.1%)	*n* = 9 (13.6%)
Widowed	*n* = 6 (5.6%)	*n* = 2 (4.9%)	*n* = 4 (6.1%)
Never married	*n* = 15 (14.0%)	*n* = 9 (22.0%)	*n* = 6 (9.1%)
Highest education			
Some high school	*n* = 1 (0.9%)	*n* = 1 (2.4%)	*N* = 0 (0.0%)
High school/GED	*n* = 4 (3.7%)	*n* = 2 (4.9%)	*n* = 2 (3.0%)
Some college/technical degree/associate’s degree	*n* = 27 (25.2%)	*n* = 14 (34.1%)	*n* = 13 (19.7%)
College degree			
Advanced degree (MS, PhD, MD)	*n* = 37 (34.6%)	*n* = 11 (26.8%)	*n* = 26 (39.4%)
	*n* = 38 (35.5%)	*n* = 13 (31.7%)	*n* = 25 (37.9%)
Concussion history	*M* = 2.9, Md = 2,	*M* = 3.4, Md = 3, IQR 1–4, SD 3.7	*M* = 2.5, Md = 1,
	IQR 0–3, SD 5.5	*n* = 7 (17.0%)	IQR 0–3, SD 6.4
0	*n* = 29 (27.1%)	*n* = 4 (9.8%)	*n* = 22 (33.3%)
1	*n* = 17 (15.9%)	*n* = 8 (19.5%)	*n* = 13 (19.7%)
2	*n* = 20 (18.7%)	*n* = 22 (53.7%)	*n* = 12 (18.2%)
3 +	*n* = 40 (37.4%)	*n* = 0 (0.0%)	*n* = 18 (27.3%)
Missing	*n* = 1 (0.9%)		*n* = 1 (1.5%)
Most recent concussion			
6–12 months ago	*n* = 0 (0.0%)	*n* = 0 (0.0%)	*n* = 0 (0.0%)
1 or more years ago	*n* = 75 (70.1%)	*n* = 32 (78.0%)	*n* = 43 (65.2%)
Missing	*n* = 32 (29.9%)	*n* = 9 (22.0%)	*n* = 23 (34.8%)
Total years of exposure to football	*M* = 4.2, SD 2.7	*M* = 5.1, SD 3.3	*M* = 3.7, SD 2.1
Total years of exposure to all contact sports	*M* = 7.5, SD 7.2	*M* = 10.6, SD 7.1	*M* = 5.6, SD 6.6
Age of first exposure to football	*M* = 11.7, Md = 12, IQR 11.0–14.0, SD 3.1	*M* = 8.4, Md = 8, IQR 8.0–12.0, SD 1.9	*M* = 13.7, Md = 13, IQR 12.0–15.0, SD 1.6

*AFE* age of first exposure, *M* mean, *Md* median, *SD* standard deviation, *IQR* interquartile range

### Univariable Analysis

Participants with AFE to football < 12 had greater exposure to football overall compared with those with AFE ≥ 12 (5.0 ± 3.3 years versus 3.7 ± 2.1 years; *p* = 0.021, *d* = − 0.75). Those in the AFE < 12 group were also significantly younger than those in the AFE ≥ 12 group (53.0 ± 13.4 versus 65.3 ± 14.2, *p* < 0.001, *d* = 0.89). However, there was no difference in the number of concussions reported between those with AFE < 12 versus AFE ≥ 12 (3.4 ± 3.7 versus 2.5 ± 6.4, respectively; *p* = 0.418, *d* = − 0.16).

When comparing groups on the basis of years of exposure to football stratified at the median years of exposure (i.e., 4 years), participants with ≥ 4 years of exposure to football had higher total GAD-7 scores compared with those with < 4 years of exposure (3.9 ± 6.0 versus 2.0 ± 2.9, *p* = 0.036, *d* = − 0.41). There were no differences in history of depression, anxiety, PTSD, PHQ-9 total score, rates of current depression or anxiety, total NSI scores, or total BC-CCI scores when comparing participants with ≥ 4 years versus < 4 years of exposure to football.

When comparing the groups on measures of psychiatric health, a greater proportion of those with AFE < 12 reported higher current PHQ-9 total scores (AFE < 12: 6.6 ± 6.5 versus AFE ≥ 12: 4.1 ± 6.0; *p* = 0.042, *d* = − 0.41) and higher current GAD-7 total scores (AFE < 12: 4.3 ± 5.6 versus AFE ≥ 12: 2.1 ± 4.1; *p* = 0.034, *d* = − 0.46), although there were no differences reported in participants’ prior histories of depression (*p* = 0.446), anxiety (*p* = 0.941), PTSD (*p* = 0.297), or substance abuse (*p* = 0.219). Additionally, there were no differences between groups in rates of neurobehavioral symptoms as measured via NSI (*p* = 0.111) or cognitive symptoms as measured via BC-CCI score (*p* = 0.267). Those with AFE < 12 reported more sleep problems (AFE < 12: 63.4% versus AFE ≥ 12: 43.9%; *p* = 0.050, *V* = 0.19). However, there were no significant differences in rates of chronic pain, migraine headaches, motor problems (dysarthria [*p* = 0.142]; balance [*p* = 0.986]; falls [*p* = 0.299]; tremor [0.991]; and any motor problem [*p* = 0.893]), or functional status (*p* = 0.311) (Table 2).

**Table 2:** Univariate comparisons on study participant demographics, general health problems, motor symptoms, functional status, and neuropsychiatric symptoms

	Age of first exposure (years)				
	< 12 (*N* = 41)	≥ 12 (*N* = 66)	*t*/*χ*^2^ value	*p*	*V*/Phi/Cohen’s *d*
Demographics					
Age	53.0 ± 13.4	65.3 ± 14.2	*t* = 4.45	** < 0.001**	*d* = 0.89
Number of prior concussions	3.4 ± 3.7	2.5 ± 6.4	*t* = − 0.82	0.418	*d* = − 0.16
Total years of football participation	5.1 ± 3.3	3.7 ± 2.1	*t* = − 3.76	** < 0.001**	*d* = − 0.75
Psychiatric symptoms					
History of depression	39.0%	31.8%	*χ*^2^ = 0.58	0.446	*V* = 0.07
History of anxiety	34.1%	34.8%	*χ*^2^ = 0.01	0.941	*V* = 0.01
History of posttraumatic stress disorder	19.5%	12.1%	*χ*^2^ = 1.09	0.297	*V* = 0.10
History of substance abuse	17.1%	9.1%	*χ*^2^ = 1.51	0.219	*V* = 0.12
PHQ-9 total score	6.6 ± 6.5	4.1 ± 6.0	*t* = − 2.06	**0.042**	*d* = − 0.41
Current depression (PHQ-9 ≥ 10)	29.3%	13.6%	*χ*^2^ = 3.92	**0.048**	*V* = 0.19
GAD-7 total score	4.3 ± 5.6	2.1 ± 4.1	*t* = − 2.16	**0.034**	*d* = − 0.46
Current anxiety (GAD-7 ≥ 10)	14.6%	6.1%	*χ*^2^ = 2.19	0.139	*V* = .14
Neurobehavioral symptoms					
Neurobehavioral symptom inventory	16.9 ± 15.7	12.3 ± 13.6	*t* = − 1.61	0.111	*d* = − 0.32
Cognitive symptoms					
BC-CCI	9.0 ± 4.2	8.1 ± 3.6	*t* = − 1.12	0.267	*d* = − 0.22
General health problems					
Sleep problems “I have had trouble falling or staying asleep.”	63.4%	43.9%	*χ*^2^ = 3.84	**0.050**	*V* = 0.19
Chronic pain “I have had pain in one or more parts of my body.”	73.2%	62.1%	*χ*^2^ = 1.38	0.240	*V* = 0.11
Migraines “I have had migraine headaches.”	14.6%	10.6%	*χ*^2^ = 0.38	0.535	*V* = 0.06
Motor symptoms					
Dysarthria “I have had difficulty with my speech, such as slurred or slow speech.”	9.8%	3.0%	*χ*^2^ = 2.16	0.142	*V* = 0.14
Balance “I have noticed difficulties or problems with my balance and my ability to walk.”	24.4%	24.2%	*χ*^2^ = 0.00	0.986	*V* = 0.002
Falls “I have lost my balance and fallen.”	4.9%	10.6%	*χ*^2^ = 1.08	0.299	*V* = 0.10
Tremor “I have a tremor (such as hand shaking).”	12.2%	12.1%	*χ*^2^ = 0.00	0.991	*V* = 0.001
Any motor problem	36.6%	37.9%	*χ*^2^ = 0.02	0.893	*V* = 0.01
Functional status			*χ*^2^ = 1.03^a^	0.311	*V* = 0.10
Independent	63.4%	72.7%			
Slightly reduced performance	26.8%	22.7%			
Definite impairment	12.2%	6.1%			
Not independent	4.9%	0.0%			
Cannot participate	2.4%	1.5%			

Bold type represents variables that reached statistical significance (*p* < 0.05)

*PHQ-9* Patient Health Questionnaire-9, *GAD-7* General Anxiety Disorder-7, *BC-CCI* British Columbia Cognitive Complaints Inventory

^a^Comparing percentage of individuals with independent functional status to nonindependent functional status

### Multivariable Analysis

Multivariable analysis examined whether AFE < 12 significantly predicted psychiatric, cognitive, neurobehavioral, and general health problems, adjusted for age, number of prior concussions, and total years of football exposure. AFE < 12 was not a significant predictor of PHQ-9 scores (*B*: 0.51, standard error, SE: 1.25, *p* = 0.682), GAD-7 scores (*B*: 0.09, SE: 0.95, *p* = 0.926), BC-CCI scores (*B*: − 0.65, SE: 0.77, *p* = 0.403), or NSI scores (*B*: − 0.56, SE: 2.93, *p* = 0.850). Regression analysis revealed that higher PHQ-9 scores were associated with younger age (*B*: − 0.13, SE: 0.04, *p* = 0.001) and a greater number of prior concussions (*B*: 0.44, SE: 0.10, *p* < 0.001). Similar patterns were noted with GAD-7 scores (age: *B*: − 0.12, SE: 0.03, *p* < 0.001; prior concussions: *B*: 0.33, SE: 0.07, *p* < 0.001), BC-CCI scores (age: *B*: − 0.08, SE: 0.02, *p* < 0.001; prior concussions: *B*: 0.26, SE: 0.06, *p* < 0.001), and NSI scores (age: *B*: − 0.31, SE: 0.09, *p* = 0.001; prior concussions: *B*: 1.04, SE: 0.23, *p* < 0.001) (Table 3).

**Table 3:** Multivariable linear regression analyses examining the effects of the age of first exposure to American Football on depression, anxiety, cognitive, and neurobehavioral symptoms in study participants

	Dependent variable: PHQ-9					
	*B*	SE	Beta	*t*	*p*	95% CI for *B*
Overall model, *F*(4,101): 8.96, *p* < 0.001, *R*^2^ = 0.26						
Age	− 0.13	0.04	− 0.31	− 3.28	**0.001**	− 0.21, − 0.05
Number of prior concussions	0.44	0.10	0.39	4.46	** < 0.001**	0.24, 0.63
Total years of football participation	− 0.09	0.21	− 0.04	− 0.43	0.667	− 0.50, 0.32
Age of first exposure	0.51	1.25	0.04	0.41	0.682	− 1.97, 3.00

	Dependent variable: GAD-7					
	*B*	SE	Beta	*t*	*p*	95% CI for *B*
Overall model, *F*(4,101): 10.79, *p* < 0.001, *R*^2^ = 0.30						
Age	− 0.12	0.03	− 0.36	− 3.86	** < 0.001**	− 0.18, − 0.06
Number of prior concussions	0.33	0.07	0.37	4.44	** < 0.001**	0.18, 0.48
Total years of football participation	0.20	0.16	0.11	1.29	0.199	− 0.11, 0.51
Age of first exposure	0.09	0.95	0.01	0.09	0.926	− 1.79, 1.97

	Dependent variable: NSI					
	*B*	SE	Beta	*t*	*p*	95% CI for *B*
Overall model, *F*(4,101): 8.83, *p* < 0.001, *R*^2^ = 0.26						
Age	− 0.31	0.09	− 0.32	− 3.37	**0.001**	− 0.50, − 0.13
Number of prior concussions	1.04	0.23	0.39	4.54	** < 0.001**	0.59, 1.50
Total years of football participation	0.18	0.48	0.03	0.37	0.715	− 0.78, 1.14
Age of first exposure	− 0.56	2.93	− 0.02	− 0.19	0.850	− 6.36, 5.25

	Dependent variable: BC-CCI					
	*B*	SE	Beta	*t*	*p*	95% CI for *B*
Overall model, *F*(4,101): 8.86, *p* < 0.001, *R*^2^ = 0.26						
Age	− 0.08	0.02	− 0.33	− 3.46	** < 0.001**	− 0.13, − 0.04
Number of prior concussions	0.26	0.06	0.38	4.33	** < 0.001**	0.14, 0.38
Total years of football participation	0.20	0.13	0.14	1.54	0.126	− 0.06, 0.45
Age of first exposure	− 0.65	0.77	− 0.08	− 0.84	0.403	− 2.17, 0.88

Bold type represents variables that reached statistical significance (*p* < 0.05)

*PHQ-9* Patient Health Questionnaire-9, *GAD-7* General Anxiety Disorder-7, *BC-CCI* British Columbia Cognitive Complaints Inventory, *NSI* Neurobehavioral Symptom Inventory, *CI* confidence interval, *SE* standard error

Regarding other health problems, AFE < 12 did not predict sleep problems (odds ratio, OR 1.28, 95% confidence interval, CI 0.49, 3.33; *p* = 0.609), chronic pain (OR 1.33, 95% CI 0.45, 3.95; *p* = 0.613), migraines (OR 0.98, 95% CI 0.25, 3.77; *p* = 0.974), any motor problems (OR 1.15, 95% CI 0.44, 3.05; *p* = 0.775), or being functionally independent (OR 0.51, 95% CI 0.18, 1.43; *p* = 0.200) in multivariable regression analyses that adjusted for age, total years of football participation, and total number of prior concussions. However, these models showed that a greater number of prior concussions were associated with a higher likelihood of having sleep problems in the past year (OR 1.30, 95% CI 1.05, 1.60; *p* = 0.017). Further, having a greater number of prior concussions was associated with a higher likelihood of having chronic pain later-in-life (OR 1.91, 95% CI 1.33, 2.73; *p* < 0.001). Younger participants had a greater likelihood of endorsing migraines (OR 0.95, 95% CI 0.91, 0.99; *p* = 0.027) (Table 4).

**Table 4:** Logistic regressions examining the effects of AFE to American Football on general health problems and functional status later in life

	Dependent variable: sleep problems						
	*B*	SE	Wald	df	*p*	OR	95% CI for OR
Overall model, *χ*^2^(4): 17.15, *p* = 0.002, Nagelkerke *R*^2^ = 0.20							
Age	− 0.02	0.02	1.64	1	0.200	0.98	0.95, 1.01
Number of prior concussions	0.26	0.11	5.73	1	**0.017**	1.30	1.05, 1.60
Total years of football participation	0.08	0.08	0.82	1	0.365	1.08	0.92, 1.27
Age of first exposure	0.25	0.49	0.26	1	0.609	1.28	0.49, 3.33

	Dependent variable: chronic pain						
	*B*	SE	Wald	*df*	*p*	OR	95% CI for OR
Overall model, *χ*^2^(4): 23.52, *p* < 0.001, Nagelkerke *R*^2^ = 0.28							
Age	0.02	0.02	0.89	1	0.345	1.02	0.98, 1.05
Number of prior concussions	0.65	0.18	12.33	1	** < 0.001**	1.91	1.33, 2.73
Total years of football participation	− 0.18	0.10	3.40	1	0.065	0.84	0.70, 1.01
Age of first exposure	0.28	0.56	0.26	1	0.613	1.33	0.45, 3.95

	Dependent variable: migraines						
	*B*	SE	Wald	*df*	*p*	OR	95% CI for OR
Overall model, *χ*^2^(4): 7.17, *p* = 0.127, Nagelkerke *R*^2^ = 0.13							
Age	− 0.05	0.02	4.92	1	**0.027**	0.95	0.91, 0.99
Number of prior concussions	0.06	0.04	2.04	1	0.153	1.06	0.98, 1.15
Total years of football participation	− 0.06	0.12	0.23	1	0.629	0.94	0.74, 1.20
Age of first exposure	− 0.02	0.69	0.00	1	0.974	0.98	0.25, 3.77

	Dependent variable: any motor problem						
	*B*	SE	Wald	*df*	*p*	OR	95% CI for OR
Overall model, *χ*^2^(4): 4.45, *p* = 0.348, Nagelkerke *R*^2^ = 0.06							
Age	0.02	0.02	2.20	1	0.138	1.02	0.99, 1.06
Number of prior concussions	0.06	0.05	1.26	1	0.262	1.06	0.96, 1.18
Total years of football participation	0.01	0.08	0.01	1	0.905	1.01	0.86, 1.18
Age of first exposure	0.14	0.50	0.08	1	0.775	1.15	0.44, 3.05

	Dependent variable: independent functional status						
	*B*	SE	Wald	*df*	*p*	OR	95% CI for OR
Overall model, *χ*^2^(4): 5.24, *p* = 0.264, Nagelkerke *R*^2^ = 0.07							
Age	− 0.01	0.02	0.39	1	0.533	0.99	0.96, 1.02
Number of prior concussions	− 0.08	0.06	1.99	1	0.159	0.92	0.83, 1.03
Total years of football participation	0.08	0.09	0.78	1	0.376	1.08	0.91, 1.28
Age of first exposure	− 0.67	0.52	1.64	1	0.200	0.51	0.18, 1.43

Bold type represents variables that reached statistical significance (*p* < 0.05)

*OR* odds ratio, *CI* confidence interval, *SE* standard error, *df* degrees of freedom

### Supplementary Analyses

Additional analyses were run that also included the eight individuals with neurological disease to attempt to account for any potential effects of healthy participant bias. Multivariable regression analysis examining predictors of PHQ-9, GAD-7, BC-CCI, and NSI with the additional eight individuals with preexisting neurological disease revealed no material changes in key regression results (i.e., no additional variables became significant predictors, and all existing significant variables remained significant; results not shown). Additional analyses were also performed that reexamined all regressions using AFE as a continuous variable instead of a binary variable; the results did not change, and AFE (as a continuous variable) was not associated with any of the aforementioned outcomes (Supplementary Table 2). Finally, additional subanalyses examined only those individuals who had a substantial exposure to football (defined here as ≥ the median of 4 years). These analyses revealed no significant difference in univariate comparisons between BC-CCI or NSI levels when stratified by AFE of 12 years (Supplementary Table 3). There were also no differences in key outcome variables (other than GAD-7 scores) when comparing participants with greater total exposure to football (i.e., those with 4 or more years of exposure versus participants with < 4 years; Supplemental Table 4). In addition, there was no change in significant predictor variables for key regression analyses examining PHQ-9, GAD-7, BC-CCI, or NSI (results not shown).

## Discussion

The current study investigated whether earlier AFE to American Football was associated with adverse brain health outcomes among a cohort of men with a history of amateur football participation. Multivariable analysis adjusted for age, number of prior concussions, and years of football exposure found no significant association between AFE < 12 and worse later-life psychiatric, neurobehavioral, cognitive, or general health problems among generally healthy adults; however, increasing number of lifetime concussions was associated with worse outcomes in these domains. The findings of this study suggest that AFE does not significantly influence later-life brain health in a cohort of generally healthy former amateur male athletes; instead, the number of prior concussions appeared to play a more critical role in predicting neuropsychiatric outcomes.

Participants who endorsed AFE < 12 did not appear to have worse health outcomes compared with those with AFE ≥ 12 across the five symptom domains considered. These findings align with existing literature suggesting that AFE does not significantly predict future health outcomes in amateur athletes. For instance, two prior studies that examined middle-aged men of age 35–55 years with a history of football participation did not find an association between AFE and depressive or cognitive symptoms [22, 23]. Moreover, shorter-term studies that analyzed the predictive value of AFE have produced similar results, specifically showing that AFE < 12 was not associated with neurocognitive functioning measured in college compared with those with AFE ≥ 12 [44]. Additionally, studies of collegiate football players have found that AFE < 12 was not associated with longer duration to symptom resolution, worse balance, worse cognitive performance, or greater psychological distress following a concussion [19]. Furthermore, a narrative review of 21 studies by Iverson et al. [45] surmised that the current body of literature does not support an association between earlier AFE and worse cognitive functioning or mental health in high school athletes, collegiate athletes, or middle-aged men. Although the majority of the current literature has studied men who play football, other studies including women rugby players [27] and those including collegiate athletes playing contact (i.e., lacrosse, wrestling, ice hockey, and soccer) or noncontact sports [21] have suggested that there is no association between earlier AFE and neurocognitive functioning, rates of depression, subjective symptoms, or quality of life. Some studies have also suggested a protective effect of earlier AFE against psychological stress and symptom severity, possibly as a result of the mental resilience and general health benefits fostered by sport participation and regular exercise [2, 18]. However, it is also possible that the lack of an association between AFE and later-life neuropsychiatric symptoms is a result of the population herein being amateur athletes, being younger, and generally healthy, all of which are protective against later-life cognitive symptoms. The data herein add to these conclusions through the addition of an analysis of mid-life individuals with amateur sport exposure to literature.

Other studies, which have primarily examined cohorts of professional football players [10, 25] and boxers [15, 46], have found associations between earlier AFE and later-life outcomes [1, 15, 26, 46, 47]. However, there are profound differences in the number and severity of head impacts experienced between amateur and professional football players that make it difficult to compare the results directly. In addition to the intrinsic differences between amateur and professional athletes, others have noted methodological issues with the Stamm et al. study that found that earlier AFE < 12 is associated with later-in-life neurocognitive deficits in NFL players [10, 11, 45, 48]. These limitations include its small sample size of symptomatic individuals, the arbitrarily chosen age of 12 years as the AFE cutoff, and the potential misinterpretation of neuropsychological tests. Further, more recent studies examining substantially larger cohorts of former NFL players [25, 49] and non-NFL players [2, 24, 44] have found no association between younger AFE and the prevalence of psychiatric or cognitive impairment later-in-life, but have instead found relationships between more seasons of play in the NFL, number of concussions, and position played in the NFL (i.e., any position except punter, kicker, or quarterback) with worse cognition-related quality of life, depression, and anxiety later in life [49]. Studies in boxers have found significant relationships between earlier AFE and later-life cognitive symptoms, brain volumes, depression, and impulsivity [15, 17, 46]. However, it is important to note that it is possible that the studies above that found no association between AFE and later-life symptoms had methodological issues analogous to those described in Stamm et al. that resulted in null findings [10].

In the current study, former football players with younger (current) age and a higher number of prior concussions had significantly worse health outcomes, specifically in the severity of psychiatric, cognitive, and neurobehavioral symptoms. Additionally, younger age was associated with a greater likelihood of current migraines, and a higher number of prior concussions were associated with an increased likelihood of sleep problems and chronic pain. Though not a part of the a priori objectives, these findings highlight potential long-term effects of concussions. Multiple studies have shown significant associations between prior concussions and higher symptom burdens at baseline [50–52] (i.e., symptoms ranging from somatic complaints such as headache, nausea, dizziness, and fatigue through to psychiatric and cognitive symptoms such as reduced concentration, attention span, and emotional lability). However, prior studies have described how many athletes have a higher symptom burden without having any significant differences in neurocognitive functioning [53, 54].

The current study is not without limitations. First, the reliance on retrospective data through self-reported patient surveys introduces the possibility of recall bias, as participants may not accurately recall details of their football or concussion history and subsequent symptomology. However, this is a general limitation of all AFE studies in the literature [2, 23, 45, 45, 49]. This study’s generalizability is constrained by the sample being predominantly white and non-Hispanic. The generalizability of this sample to the general population is also constrained by the use of self-report surveys for the outcome measures. The proportion of general health problems is similar to national averages [55, 56], yet the proportion with a college education (70%) is higher, though this is a common finding in other survey studies [57]. Furthermore, the current study only considered football players; therefore, the results may not be applicable to other contact sports. Additionally, because of the smaller sample size, univariate comparisons were not statistically feasible for some demographic variables (e.g., for race, which only had *n* = 3 Black/African American individuals in the AFE ≥ 12 group). Moreover, this study included a relatively small number of participants and excluded a small number of individuals (*n* = 8) with preexisting neurological disease, which may predispose the study to healthy participant bias. Further, this study did not account for exposure from other contact sports. Thus, some participants may have had exposure to football and one or more additional contact sports. However, the percentage of participants who reported exposure to any single contact sport other than football was relatively minimal, with martial arts having the highest number of participants reporting exposure (*n* = 9, 8.4%). Finally, the results herein may differ if the population studied were less healthy, older, or played professional sports. Future studies should analyze prospective data to mitigate recall bias, diversify participant demographics to enhance generalizability, and extend investigations to a broader range of contact sports to improve external validity.

## Conclusions

In a cohort of generally healthy mid-to-later-life adults with exposure to amateur-level American Football, younger exposure to football was not significantly associated with psychiatric or neurobehavioral symptoms, cognitive difficulties, general health problems, motor symptoms, or functional status in adjusted models. Interestingly, rather than AFE to football, a greater number of prior concussions was significantly associated with worse psychiatric, cognitive, and neurobehavioral symptoms, highlighting the potential impact that repeated concussions may have on health outcomes rather than simply exposure to football. Prospective, longitudinal studies with older individuals from diverse backgrounds and more health comorbidities are needed to increase the external validity of the results presented herein.

## Supplementary Information

Below is the link to the electronic supplementary material.

Supplementary file1 (PDF 156 kb)

## Declarations

### Data availability

Data are available to qualified investigators on reasonable request.
