# Supplementary material for: The Association Between Age of First Exposure to American Football at a Young Age and Later-Life Health Issues in Healthy, Community-Dwelling Adults
Source: Sports Med. 2025 May 14;55(10):2613–25. doi: 10.1007/s40279-025-02239-w (PMC12513914; doi:10.1007/s40279-025-02239-w)
Supplement: Supplementary file 1 — Supplementary file1 (PDF 156 kb) [file 40279_2025_2239_MOESM1_ESM.pdf]

Title: Age of first exposure to football and later-life health issues in community-dwelling adults

Journal Name: *Sports Medicine*

Authors:

Grant H. Rigney, MSc, MSc[1,2]
John E. Dugan, BA[2,3]
Anthony Bishay, BA[2,4,5]
Soren Jonzzon, MD[2,5]
Jacob Jo, BA[2,4,5]
Kristen L. Williams, MS, LAT, ATC[2,5]
Scott L. Zuckerman, MD, MPH[2,5]
Douglas P. Terry, PhD[2,5]

Affiliations:
[1]Harvard Medical School, Boston, MA, USA
[2]Vanderbilt Sport Concussion Center, Vanderbilt University Medical Center, Nashville, TN, USA
[3]The University of Tennessee Health Science Center, Memphis, TN, USA
[4]Vanderbilt University School of Medicine, Nashville, TN, USA
[5]Department of Neurological Surgery, Vanderbilt University Medical Center, Nashville, TN

Corresponding Author:
Douglas P. Terry, PhD, douglas.terry@vumc.org
1500 21st Ave S, Village at Vanderbilt, Suite 4300, Nashville, TN, 37212
Vanderbilt Sport Concussion Center, Department of Neurological Surgery
Vanderbilt University Medical Center

Supplement Table 1

Functional Status Question

With regard to your:
- Job
- Household responsibilities
- Family, social, and community roles

Which of the follow best describes your current level of functioning? (Select one. For definitions, see below)

☐	I am independent.<br>• I am able to engage in hobbies and intellectual activities at my usual levels.<br>• I am fully independent in my activities.
☐	I have slightly reduced performance.<br>• I have slight problems in hobbies and intellectual interests.<br>• I am mostly independent but may be more challenged in some *advanced activities.***<br>• I am fully independent in *basic activities.**
☐	I have definite impairment of *advanced activities.***<br>• I am engaged in some home, family, social, and community activities.<br>• I abandon more difficult activities.<br>• I need cues for some *basic activities.**
☐	I am not independent but can be taken to some functions outside the home.<br>• I can do only simple chores.<br>• I have very restricted interests.<br>• I need assistance with *basic activities.**
☐	I cannot participate in functions outside the home.<br>• I have impaired *basic activities.**<br>• I am not independent with self-care.<br>• I am frequently incontinent.

***Basic activities are related to:**	****Advanced activities are related to:**
Personal hygiene and grooming (e.g., brushing, combing, styling hair)	Managing money (e.g., paying bills, completing taxes)
Toilet hygiene (e.g., getting to the toilet, cleaning oneself, and getting back up)	Cleaning and maintaining the house
Bathing/showering	Preparing meals
Dressing	Shopping for groceries and necessities
Self-feeding	Medication management
Functional mobility (e.g., ability to walk, get in and out of bed, get into and out of a chair)	Transportation within the community (e.g., driving, using public transportation)
	Using the telephone (mobile or landline)

Supplemental Table 2: Multivariable linear regression analyses examining the effects of age of first exposure (as a linear variable) to football on depression, anxiety, cognitive, and neurobehavioral symptoms.

**Dependent Variable: PHQ-9**						
	**B**	**SE**	**Beta**	**t**	**p**	**95% CI for B**
Age	-0.15	0.04	-0.36	-3.80	**<.001**	-0.23, -0.07
Number of Prior Concussions	0.44	0.10	0.39	4.51	**<.001**	0.25, 0.64
Total Years of Football Participation	-0.02	0.21	-0.01	-0.11	.917	-0.44, 0.39
Age of First Exposure	0.15	0.20	0.07	0.71	.479	-0.26, 0.55
**Dependent Variable: GAD-7**						
	**B**	**SE**	**Beta**	**t**	**p**	**95% CI for B**
Age	-0.13	0.03	-0.40	-4.30	**<.001**	-0.19, -0.07
Number of Prior Concussions	0.33	0.07	0.38	4.48	**<.001**	0.19, 0.48
Total Years of Football Participation	0.25	0.16	0.14	1.59	.115	-0.06, 0.56
Age of First Exposure	0.14	0.15	0.09	0.93	.356	-0.16, 0.45
**Dependent Variable: NSI**						
	**B**	**SE**	**Beta**	**t**	**p**	**95% CI for B**
Age	-0.36	0.09	-0.37	-3.88	**<.001**	-0.54, -0.18
Number of Prior Concussions	1.05	0.23	0.40	4.60	**<.001**	0.60, 1.50
Total Years of Football Participation	0.35	0.48	0.06	0.72	.476	-0.61, 1.31
Age of First Exposure	0.63	0.47	0.13	1.33	.187	-0.31, 1.57
**Dependent Variable: BC-CCI**						
	**B**	**SE**	**Beta**	**t**	**p**	**95% CI for B**
Age	-0.09	0.02	-0.36	-3.83	**<.001**	-0.14, -0.05
Number of Prior Concussions	0.26	0.06	0.37	4.37	**<.001**	0.14, 0.38
Total Years of Football Participation	0.23	0.13	0.16	1.80	.074	-0.02, 0.48
Age of First Exposure	0.20	0.12	0.16	1.62	.108	-0.05, 0.45

Supplemental Table 3: Sub-analysis examining univariate comparisons of participants with more than the median number of years of exposure to football (i.e., ≥4 years), stratified by AFE of 12.

	**<12 (n=25)**	**≥12 (n=30)**	**t/X^2 value**	**P value**	**V/Phi/Cohen's d**
History of Depression	32.0%	23.3%	X^2=0.52	.472	V=0.09
History of Anxiety	32.0%	26.7%	X^2=0.19	.665	V=0.06
History of Post-Traumatic Stress Disorder	24.0%	6.7%	X^2=3.30	.069	V=0.25
History of Substance Abuse	8.0%	6.7%	X^2=4.07	.254	V=0.27
PHQ-9 Total Score	6.5±7.1	4.5±7.5	t=-1.0	.312	d=-0.28
Current Depression (PHQ-9≥10)	24.0%	13.3%	X^2=1.0	.307	V=.14
GAD-7 Total Score	4.8±6.4	3.1±5.7	t=-1.1	.299	d=-0.28
Current Anxiety (GAD-7≥10)	16.0%	13.3%	X^2=0.08	.780	V=0.04
Neurobehavioral Symptom Inventory (NSI)	17.6±16.8	14.1±17.9	t=-0.7	.457	d=-0.20
BC-CCI	9.5±4.7	8.6±4.3	t=-0.7	.490	d=-0.19

Supplemental Table 4: Sub-analysis comparing key outcome variables between participants with more or less than 4 years of exposure to football.

	**<4 (n=52)**	**≥4 (n=55)**	**t/X^2 value**	**P value**	**V/Phi/Cohen's d**
PHQ-9 Total Score	4.7±5.0	5.4±7.3	t=0.56	.577	d=0.11
Current Depression (PHQ-9≥10)	21.1%	18.2%	X^2=0.15	.699	V=0.04
GAD-7 Total Score	1.9±2.9	3.9±6.0	t=2.13	**.036**	d=0.41
Current Anxiety (GAD-7≥10)	3.8%	14.5%	X^2=3.61	.057	V=0.18
Neurobehavioral Symptom Inventory (NSI)	12.3±10.6	15.7±17.3	t=1.23	.221	d=0.24
BC-CCI	7.8±3.0	9.0±4.5	t=1.60	.113	d=0.31
